# The Effectiveness Assessment of Agricultural Subsidy Policies on Food Security: Evidence from China’s Poverty-Stricken Villages

**DOI:** 10.3390/ijerph192113797

**Published:** 2022-10-24

**Authors:** Chengyou Li, Zhouhao Sha, Xiaoqin Sun, Yong Jiao

**Affiliations:** 1 The Center for Economic Research, Shandong University, Jinan 250100, China; 2 School of Finance, Shandong University of Finance and Economics, Jinan 250014, China; 3 School of Statistics and Mathematics, Shandong University of Finance and Economics, Jinan 250014, China; 4 College of Economics & Management, Shandong University of Science and Technology, Qingdao 266590, China

**Keywords:** agricultural subsidy, effect assessment, poverty areas, panel data

## Abstract

This paper builds a theoretical model based on a representative peasant household in the neoclassical model, comprehensively considers three types of farmer households in China, and evaluates the effects of the agricultural subsidy policy under equilibrium conditions. Based on the two bottom lines of guaranteeing China’s grain security and ensuring no large-scale return to poverty, this paper uses 2010, 2012, and 2014 tracking survey data from the Mutual Aid Fund for Poverty-Stricken Villages in China to construct an econometric model to evaluate agricultural subsidy effectiveness from the three aspects of farmers’ total sown area, total grain output, and total income. The research draws the following conclusions: (1) Agricultural subsidies can significantly increase the sown area, grain production, and total income of farmers in poverty-stricken areas, which is conducive to improving the farmers’ comprehensive capacity for grain production as well as income, and this conclusion remains valid after performing a series of robustness tests and solving endogeneity problems. (2) The effects of the agricultural subsidy policy are affected by natural conditions, economic development levels, and functional orientation of grain production in different regions, and they have divergent influences on farmers’ total sown area, total output, and total income. (3) Agricultural subsidies boost farmers’ willingness to cultivate grain, reduce land abandonment, and increase the total sown area, total output, and total grain income. The willingness to cultivate grain is an important mechanism that affects the effectiveness of the agricultural subsidy policy.

## 1. Introduction

Agricultural subsidies are one of the most effective policies for governments around the world to support and protect agricultural development, and China is no exception [1,2,3]. Since 2004, the Chinese government has gradually established an agricultural subsidy system to issue subsidies for purchasing fine seeds, directly subsidize grain growers, and distribute general subsidies for purchasing agricultural supplies [4,5]. The purpose of China’s agricultural subsidy policy is to raise farmers’ enthusiasm for planting grain, ensure food security, and increase farmers’ income [6]. Therefore, after more than ten years of hard work, the following questions are raised. Has China truly achieved the main goal of implementing this agricultural subsidy policy? What is its mechanism? How should it be optimized in the future? Can it provide a reference for other countries and regions? Consequently, a detailed assessment of the effectiveness of the agricultural subsidy policy is required to answer these questions.

Since the implementation of the agricultural subsidy policy, scholars have conducted fruitful research on the effect of agricultural subsidy policies but have not reached consensus [7,8,9]. From the perspective of agricultural subsidies’ effects on farmers’ initiative for growing grain, some scholars found that they have a marginal incentive effect and play a positive role for farmers who are uncertain about participating in grain planting, improving farmers’ enthusiasm for grain planting and increasing the sown area [10]. Meanwhile, some studies further concluded that agricultural subsidies not only help stimulate farmers’ enthusiasm for cultivating grain but also have a positive impact on grain production [11,12]. A few scholars have also used grain productivity to measure the yield-increasing effect of agricultural subsidy policies [13,14,15]. For example, Zhang et al. [6] used the Data Envelopment Analysis (DEA) method to construct a Malmquist index model based on the statistical data of China’s main grain-producing areas from 2008 to 2017 to measure grain production efficiency. They then analyzed the relationship between agricultural subsidies and grain productivity and proposed that the former promoted the steady growth of the latter. In addition, some scholars also constructed various forms of econometric models based on data from diverse sources in terms of increasing farmers’ income and estimated the impact of agricultural subsidies on farmers’ income. They presented that agricultural subsidies boost the income of farmers who plant grain, effectively reducing the income distribution gap [16,17,18,19].

However, some scholars hold different views on the above research [5,20]. Some believe that the government’s agricultural subsidies cannot effectively raise the enthusiasm of farmers for grain production. Even if the grain subsidies are continuously raised, the impact on farmers’ enthusiasm for grain production is absolutely limited [21]. Some studies also claim that agricultural subsidy policies cannot lead to the expansion of grain cultivation or the increase in grain production and have only a slight effect on rural development [22,23]. Moreover, some scholars refute findings from the perspective of income and find that agricultural subsidies have no impact on the promotion of farmers’ income [24]. Not only that, but with the continuous emergence of new types of agricultural business entities, there is often a mismatch of resources between the subsidizer and the subsidized party, which not only reduces the farmers’ enthusiasm to grow grain but also deteriorates grain production and farmers’ income [20]. In addition, many scholars have pointed out that the effects of agricultural subsidy policies differ significantly at different stages. In the early stage of the implementation, the policies indeed stimulate farmers’ enthusiasm for planting grain, expand the sown area, and further increase grain output and farmers’ income, while in the long term, this effect shows a decreasing trend until it disappears [25,26].

In terms of the mechanism of the effects of agricultural subsidy policies, scholars put forward that agricultural subsidies will change the amount of agricultural inputs, such as fertilizers, seeds, pesticides, and agricultural machinery, which will contribute to the improvement of grain output and farmers’ income [6,27]. Agricultural subsidies can also promote the remuneration of agricultural labor relative to non-agricultural labor and encourage farmers to engage in agriculture rather than work as migrant workers [28,29]. Moreover, agricultural subsidies will greatly stimulate the motivation of farmers to invest in human capital, such as hiring other farmers to participate in agriculture activities or attending training classes to master more skills, all of which are conducive to increasing food production and farmers’ income [30]. Apart from that, from the perspective of credit constraints, some scholars conclude that agricultural subsidies can alleviate farmers’ liquidity limitations, reduce land vacancy, and increase grain production and farmers’ income [10,16].

Summarizing the above literature, it is evident that previous studies have drawn abundant conclusions, laying a solid foundation and providing a valuable reference for the research of this paper. However, there are still some deficiencies that require further investigation. First, previous related research on the effect of agricultural subsidy policies has mainly been based on practical observation and logical analysis, and most of it has been analyzed from the perspective of macroeconomic policies, while few scholars have launched studies by constructing theoretical models. Even though some scholars have used theoretical models, the applicability of such models is limited, and theoretical analyses considering various types of peasant households in China have been scarce; thus, these need to be further supplemented and improved. Second, some scholars have conducted empirical research on the effects of agricultural subsidy policies from various perspectives, but few have analyzed the effect of the agricultural subsidy policy in poor areas in China. Furthermore, studies based on panel data and large samples of micro-farmer households have been limited. Third, some scholars have used a variety of measurements to evaluate the effect of agricultural subsidy policies, but there have been few comprehensive evaluations in terms of sown area, grain yield, and grain income, and scholars have seldom paid attention to the critical role that farming willingness plays in agricultural subsidies. Fourth, some studies have not fully addressed the possible endogeneity problems in the econometric model and have not proceeded with a comprehensive analysis of the robustness and heterogeneity of the estimation process, which may lead to a biased regression result. Fifth, various studies have put forward a wealth of countermeasures and suggestions based on the effectiveness of agricultural subsidy policies, but not many have been aimed at poverty-stricken areas or put forward methods to solve the problems of food security and farmers’ low income in poor areas from the perspective of agricultural subsidies.

Therefore, based on the above existing research results, this paper derived the effects of the agricultural subsidy policy under equilibrium conditions. These predictions were then tested using the tracking survey data of China’s poverty-stricken villages’ mutual aid fund project. In particular, an econometric model was consequently constructed to evaluate the policy effectiveness from the three aspects of farmers’ total sown area, total output, and total income based on the two bottom lines of guaranteeing China’s grain security and ensuring no large-scale return to poverty. The research draws the following conclusions: Agricultural subsidies can significantly increase the sown area, grain production, and total income of farmers in poverty-stricken areas, which is conducive to improving the farmers’ comprehensive capacity for grain production as well as income. However, this result is affected by natural conditions, economic development levels, and functional orientation of grain production in different regions. Furthermore, the willingness to cultivate grain is an important mechanism that affects the effectiveness of the agricultural subsidy policy.

Compared with previous studies, the main contributions are as follows. First, a theoretical model is established based on a typical farmer family in the neoclassical model, considering three types of Chinese farmers, to theoretically analyze the effect of the agricultural subsidy policy on various types of rural households. Second, we use the tracking panel data of 1116 farmers from 2010, 2012, and 2014, covering 50 impoverished villages in 10 counties and cities in the five provinces of Henan, Shandong, Sichuan, Hunan, and Gansu, to analyze the effectiveness of the agricultural subsidy policy in poor areas in China. The data are highly representative, and the conclusions obtained are remarkably convincing. Third, this paper comprehensively and objectively evaluates the impact of agricultural subsidies. From the perspective of farming willingness, we analyze the mechanism of the agricultural subsidy policy by investigating the changes in the proportion of idle land and broadening the research directions on the effectiveness of the agricultural subsidy policy. Fourth, the instrumental variable method is used to solve the possible endogeneity problems in the process of the empirical test. Additionally, a multi-angle robustness test and a multi-level heterogeneity analysis are carried out in the econometric model to ensure the reliability of the research conclusions. Fifth, according to the conclusions drawn by the theoretical analysis and empirical testing, corresponding countermeasures and suggestions are proposed based on the Chinese experience to provide a reference to the poor areas throughout the world facing food crisis and extreme poverty.

## 2. Theoretical Analysis

We establish the theoretical model based on a typical peasant household in the new classical model and assume that there are two sectors in the region where the representative peasant household is located: the agricultural sector and the non-agricultural sector. Among them, the agricultural sector can be further divided into the food crop and non-food crop production sectors. Typically, farmers need to allocate labor and other means of production between food crops, non-food crops, and non-agricultural production.

Before constructing the theoretical model, we propose the following assumptions:

(1) T represents the total labor time of the peasant household, $l_{g}$ is the labor time of the food crop production, $l_{o}$ is the labor time of the non-food crop production, and $l_{na}$ is the labor time of non-agricultural production.
(2) The utility of peasant households comes from two parts: consumption and leisure.
(3) The production function of food crops is expressed as $f_{g}=A_{g}l_{g}{}^{\alpha }k_{g}{}^{1-\alpha }$, and the production function of non-food crops is expressed as $f_{o}=A_{o}l_{o}{}^{\beta }k_{o}{}^{1-\beta }$.
(4) Without inputting other means of production, labor is all the non-agricultural production needs, and its output is $f_{na}=l_{na}{}^{\gamma }$.
(5) Assuming that the price of food crops is $p_{g}$, the price of non-food crops is $p_{o}$, and the wage of the non-agricultural production sector is ω, the income of farmers in the food crop production sector can be expressed as $Y_{g}=p_{g}A_{g}l_{g}{}^{\alpha }k_{g}{}^{1-\alpha }$, that in the non-food crop production sector is $Y_{o}=p_{o}A_{o}l_{o}{}^{\beta }k_{o}{}^{1-\beta }$, and that in the non-agricultural production sector is $Y_{na}=\omega l_{na}{}^{\gamma }$.

According to Assumption (2), we set the utility function of farmers based on the household utility function constructed by Becker [31] as Equation (1):

(1) $$\underset{l_{g},l_{o},l_{na}}{\max}U=\ln C+\varphi \ln(T-l_{g}-l_{o}-l_{na})$$

In Equation (1), C represents the commodities consumed by farmers, and lnC is the utility obtained by farmers from commodity consumption. Moreover, $T-l_{g}-l_{o}-l_{na}$ represents the leisure time of farmers, $\ln(T-l_{g}-l_{o}-l_{na})$ is the utility obtained by farmers from leisure, and φ≥0 is the conversion coefficient between consumption and leisure.

Farmers’ budgets will be constrained when they carry out a series of economic activities. Therefore, combined with Assumptions (3)–(5), the budget constraints of farmers can be expressed as Equation (2):

(2) $$\begin{array}{l} s.t.\\ C\leq Y,Y=p_{g}A_{g}l_{g}{}^{\alpha }k_{g}{}^{1-\alpha }+p_{o}A_{o}l_{o}{}^{\beta }k_{o}{}^{1-\beta }+\omega l_{na}{}^{\gamma }-c_{g}k_{g}-c_{o}k_{o} \end{array}$$

In Equation (2), $c_{g}$ is the price of food crops as means of production, $c_{g}k_{g}$ represents the cost of food crops as means of production, $c_{o}$ is the price of non-food crops as means of production, and $c_{o}k_{o}$ represents the cost of non-food crops as means of production. Equation (2) indicates that in the absence of borrowing, farmers’ total consumption cannot exceed their total disposable income. The total disposable income is the distribution of farmers’ means of production between the agricultural sector and the non-agricultural sector, such as labor, time, and land; that is, the income allocated to the production of food crops, non-food crops, and non-agricultural production minus the input cost of agricultural production materials, which is the cost of food crops and non-food crops as means of production. Since the machinery and equipment in the non-agricultural production sector are provided by the manufacturers, farmers are not directly responsible for this part.

In China, the target of agricultural subsidies is farmers who cultivate the land, reflecting the principle of “Who farms, who subsidized”. Therefore, this paper divides farmers into three categories: (1) farmers who only engage in the cultivation of food crops, (2) farmers who engage in the cultivation of both food crops and non-food crops, and (3) “Part work and part cultivate” farmers; that is, those who engage in both agricultural and non-agricultural activities. Next, we will analyze these three types of farmers separately.

### 2.1. Farmers Only Engaged in Food Crop Cultivation

For farmers who only engage in food crop cultivation, since non-food crop production and non-agricultural activities are not included, the utility function of this type of farmer (Type (1)) can be expressed as Equation (3):

(3) $$\underset{l_{g},k_{g}}{\max}U=\ln C+\varphi \ln(T-l_{g})$$

Notably, the agricultural subsidy issued to Type (1) farmers according to the agricultural output is the first type of subsidy, which can directly increase the income brought by the farmers’ grain production. We denote $\pi_{1}$ as the agricultural subsidy issued according to the output; then, the income from grain production can be denoted as $(p_{g}+\pi_{1})A_{g}l_{g}{}^{\alpha }k_{g}{}^{1-\alpha }$. On the other hand, there is another subsidy based on the planting area. If the cultivated area is S, then the subsidy is σS. Since land is a part of $k_{g}$, as long as the production structure remains unchanged, the ratio of S to $k_{g}$ does as well, and the subsidy is equivalent to $\pi_{2}k_{g}$. Thus, the constraint function after adding agricultural subsidies can be expressed as Equation (4):

(4) $$\begin{array}{l} s.t.\\ C\leq Y,Y=(p_{g}+\pi_{1})A_{g}l_{g}{}^{\alpha }k_{g}{}^{1-\alpha }-(c_{g}-\pi_{2})k_{g} \end{array}$$

Combining Equations (3) and (4), we obtain the following results:$\frac{\partial k_{g}^{*}}{\partial \pi_{1}}$ > 0, $\frac{\partial k_{g}^{*}}{\partial \pi_{2}}$ > 0, $\frac{\partial f_{g}^{*}}{\partial \pi_{1}}$ > 0, $\frac{\partial f_{g}^{*}}{\partial \pi_{2}}$ > 0, $\frac{\partial l_{g}^{*}}{\partial \pi_{1}}$ = 0, $\frac{\partial l_{g}^{*}}{\partial \pi_{2}}$ = 0. These results mean that agricultural subsidies have no significant impact on the time spent on grain planting for farmers who only engage in the cultivation of food crops, and mainly influence the output by increasing the input of means of production $k_{g}$ and thus affect the income of farmers.

### 2.2. Farmers Engaged in Both Food Crop and Non-Food Crop Cultivation

For Type (2) farmers who plant both food and non-food crops, considering that they only engage in food and non-food production activities in the agricultural sector rather than non-agricultural activities, the objective function of this type of farmer can be expressed as Equation (5):

(5) $$\underset{l_{g},l_{o},k_{g},k_{o}}{\max}U=\ln C+\varphi \ln(T-l_{g}-l_{o})$$

When agricultural subsidies are issued to Type (2) farmers, in addition to the similarities with Type (1) farmers, they can also add the opportunity cost of planting non-food crops. That is, $c_{o}k_{o}$ increases to $(c_{o}+\pi_{3})k_{o}$, and the constraint becomes Equation (6):

(6) $$\begin{array}{l} s.t.\\ C\leq Y,Y=(p_{g}+\pi_{1})A_{g}l_{g}{}^{\alpha }k_{g}{}^{1-\alpha }+p_{o}A_{o}l_{o}{}^{\beta }k_{o}{}^{1-\beta }-(c_{g}-\pi_{2})k_{g}-(c_{o}+\pi_{3})k_{o} \end{array}$$

Combining Equations (5) and (6), we obtain the following results: $\frac{\partial k_{g}^{*}}{\partial \pi_{1}}$ > 0, $\frac{\partial k_{g}^{*}}{\partial \pi_{2}}$ > 0, $\frac{\partial f_{g}^{*}}{\partial \pi_{1}}$ > 0, $\frac{\partial f_{g}^{*}}{\partial \pi_{2}}$ > 0, $\frac{\partial k_{o}^{*}}{\partial \pi_{3}}$ < 0, $\frac{\partial f_{o}^{*}}{\partial \pi_{3}}$ < 0. These results reveal that agricultural subsidies enhance the willingness of this type of farmer to cultivate crops. Specifically, agricultural subsidies principally increase the grain production input $k_{g}$ for Type (2) farmers and reduce the input of other crops *k*_0_ to stimulate grain output and thus enhance the grain income of farmers.

### 2.3. “Part Work and Part Cultivate” Farmers Engaged in Both Crop Cultivation and Non-Agricultural Activities

For Type (3) farmers engaged in both crop cultivation and non-agricultural activities, the objective function can be expressed as Equation (7):

(7) $$\underset{l_{g},l_{na},k_{g}}{\max}U=\ln C+\varphi \ln(T-l_{g}-l_{na})$$

When governments distribute agricultural subsidies to Type (3) farmers, similar to Type (1) farmers, the constraints can be rewritten as Equation (8):

(8) $$s.t.C\leq Y,Y=(p_{g}+\pi_{1})A_{g}l_{g}{}^{\alpha }k_{g}{}^{1-\alpha }+\omega l_{na}{}^{\gamma }-(c_{g}-\pi_{2})k_{g}$$

Combining Equations (7) and (8), we obtain the following results: $\frac{\partial l_{g}^{*}}{\partial \pi_{1}}$ > 0, $\frac{\partial l_{g}^{*}}{\partial \pi_{2}}$ > 0, $\frac{\partial k_{g}^{*}}{\partial \pi_{1}}$ > 0, $\frac{\partial k_{g}^{*}}{\partial \pi_{2}}$ > 0, $\frac{\partial f_{g}^{*}}{\partial \pi_{1}}$ > 0, $\frac{\partial f_{g}^{*}}{\partial \pi_{2}}$ > 0. We conclude that agricultural subsidies significantly increase the time $l_{g}$ and input $k_{g}$ of planting grain, reduce the area of idle land, and enhance grain output and farmers’ income. Moreover, $\frac{\partial l_{na}}{\partial \pi_{1}}<0$, $\frac{\partial l_{na}}{\partial \pi_{2}}<0$, $\frac{\partial f_{na}}{\partial \pi_{1}}<0$, and $\frac{\partial f_{na}}{\partial \pi_{2}}<0$ can be obtained as well, which also implies that agricultural subsidies will substitute for non-agricultural working time and non-agricultural output to a certain degree. With the improvement of the agricultural subsidy system, farmers’ willingness to engage in cultivation is raised, and their intentions to participate in non-agricultural sectors are reduced simultaneously, which encourages more farmers to work in specialized agricultural production activities.

In short, the analysis of the different effects of agricultural subsidy policy on the above three types of farmers clearly shows that agricultural subsidies have a positive impact on grain planting input, grain output, and grain income for the above three types of farmers. Therefore, we draw Inference 1 of this paper:

**Inference** **1:** *Agricultural subsidies can promote farmers’ grain input, grain output, and grain income.*

In addition, according to the above theoretical model, the relationship between agricultural subsidies and the grain planting input of the three types of farmers is significantly positive, especially the input of means of production $k_{g}$ for cultivating crops. Generally speaking, the land is the primary input in means of production. The increase in agricultural subsidies enhances farmers’ willingness to plant crops. Consequently, they want to scale up the input of land as a means of production, contributing to the increase in sown area and the decrease in the area of idle land, ultimately stimulating both grain output $f_{g}=A_{g}l_{g}{}^{\alpha }k_{g}{}^{1-\alpha }$ and grain income. Hence, we draw Inference 2 of this paper:

**Inference** **2:** *Agricultural subsidies increase farmers’ grain input, grain output, and grain income by reducing the area of idle land. Farming willingness is an important mechanism that influences the effect of the agricultural subsidy policy.*

## 3. Data sources, Variable Selection, and Model Construction

### 3.1. Data Sources

This paper uses 2010, 2012, and 2014 tracking survey data from the Mutual Aid Fund for Poverty-Stricken Villages in China obtained by stratified sampling, which has strong authenticity, representativeness, and typicality. First, according to the differences in the topographic features and regional economic development levels in China, five representative provinces were chosen as the target provinces: Shandong, Henan, Hunan, Gansu, and Sichuan. Second, two poverty-stricken counties were identified in each of the five target provinces. Third, from each of the 10 poor counties in this survey, five poor villages were selected as sample villages. Finally, from each of the 50 sample villages, 30 peasant households were chosen as the research objects, yielding a total of 1500 households. The content of this survey mainly included the fundamental characteristics of the head of the household, their family background, the basic situation of agricultural production, whether they were participating in the mutual aid project, the basic situation of their local village, and so on. After eliminating invalid samples from the original data, 1116 peasant households were included as valid samples.

### 3.2. Variable Selection

#### 3.2.1. Dependent Variables

Three comprehensive indicators of total sown area, total grain output, and total grain income were used to measure the effects of the agricultural subsidy policy. Specifically, the total sown area and total grain output were selected to measure the farmers’ comprehensive capacity for grain production, while the total grain income was chosen to measure the income level of farmers. Meanwhile, we tried to use these three comprehensive indicators to further reflect whether China has firmly adhered to the two bottom lines of guaranteeing national food security and ensuring no large-scale return to poverty. In addition, for the definition of grain, there are broad and narrow concepts. In the broadest sense, grain refers to all kinds of food crops, such as wheat, corn, soybeans, and potatoes. Conversely, in the narrow sense, the concept of grain usually refers to cereal crops, such as wheat, corn, and rice. In this paper, we define grain in accordance with its generalized concept. In addition, to eliminate the effect of heteroscedasticity on the estimated results, the logarithmic transformation was carried out on the dependent variables in the regression.

#### 3.2.2. Independent Variable

The total amount of agricultural “triple subsidy” in China received by farmers is the core explanatory variable of this paper, also known as the protection subsidy for the productivity of the cultivated land. Specifically, the “triple subsidy” for agriculture includes subsidies for purchasing fine seeds, direct subsidies to grain growers, and general subsidies for purchasing agricultural supplies. All these subsidies are directly handed out to farmers through “smart cards.”

#### 3.2.3. Control Variables

Following the principle of choosing variables that are as exogenous as possible, the control variables in this paper were selected from three aspects. Accordingly, in terms of the basic characteristics of the head of household, variables such as age, education level, health status, and cadre status were selected. In terms of their family background, the size of the household labor force, whether the family members have opportunities to go out as migrant workers, the value of household production and operation fixed assets, the expenditure on household gift-giving for interpersonal interaction, whether the family can borrow money from the private sector, and whether the family can gain access to loans from financial institutions were selected. Moreover, based on the characteristics of the household heads’ local villages, we chose variables such as whether the village is a credit village assessed by credit cooperatives and the frequency of “two rural committee meetings” in the villages as the control variables.

#### 3.2.4. Mediator Variables

The primary purpose of the mediator effect is to examine the role of farmers’ willingness to cultivate land in the impact of the agricultural subsidy policy. Therefore, combined with the research objects and survey data, we selected the proportion of idle land, which is the ratio of the area of wasteland in all farmland to the area of farmland contracted by the farmers, to measure farmers’ willingness to cultivate grain.

The descriptive statistics of the main variables in this study are presented in Table 1.

### 3.3. Model Construction

Based on the previous studies, the following measurement models are constructed to investigate the effects of the agricultural subsidy policy:

(9) $$y_{it}=\beta_{10}+\beta_{11}subsidy_{it}+\Sigma \beta_{1k}X_{k,it}+\varepsilon_{it}$$

In Equation (9), $y_{it}$ is the three indicators of the dependent variables, which are the total sown area, total grain output, and total grain income of household i in year t. $\beta_{10}$ represents the intercept term, $subsidy_{it}$ represents the amount of the agricultural subsidy, and $\beta_{11}$ is its coefficient. $X_{k,it}$ represents a series of control variables, and $\beta_{1k}$ represents the coefficient of these variables. $\varepsilon_{it}$ is the random error.

To further examine the role of farming willingness in the effect of the agricultural subsidy policy, referring to the methods of Li et al. [23] and Zheng et al. [32], we continue to construct the following equation to test the mediation effect:

(10) $$med_{it}=\beta_{20}+\beta_{21}subsidy_{it}+\Sigma \beta_{2k}X_{k,it}+\varepsilon_{it}$$

(11) $$y_{it}=\beta_{30}+\beta_{31}subsidy_{it}+\beta_{32}med_{it}+\Sigma \beta_{3k}X_{k,it}+\varepsilon_{it}$$

In Equations (10) and (11), $med_{it}$ represents the mediator variable, which is an indicator of the farmers’ willingness to cultivate grain, measured by the ratio of land vacancy. Since the other variables in Equations (10) and (11) are as specified for Equation (9) described above, no detailed description is given here.

## 4. Results Estimation, and Robustness Tests

### 4.1. Results Estimation

Based on the results of the Hausman test, we used a fixed-effect model to analyze the effects of the agricultural subsidy policy in the benchmark regression. The specific results are provided in Table 2. Table 2 reveals that the impact of agricultural subsidies on the total sown area and that on the total grain output of farmers are both positive at the 1% significance level. The agricultural subsidies also have a positive effect on the total grain income of farmers at the 5% significance level. Consistent with the conclusions obtained by the theoretical model, the regression results indicate that agricultural subsidies can enhance farmers’ enthusiasm for grain cultivation, improving farmers’ grain output and grain income. In other words, in poverty-stricken areas of China, agricultural subsidies can improve the disposable income of farmers and motivate them to participate in cultivation, thus increasing the sown area and ultimately farmers’ grain production and income. Influence 1 of this paper is confirmed. This result is largely consistent with other scholars’ assessments of the effects of agricultural subsidy policies in some regions of China during the same period [18,20]. However, unlike other scholars’ studies, this paper is a comprehensive and objective assessment of the effects of agricultural subsidies on poor areas in China from three aspects: total sown area, total output and total income of farm households. The results of this paper not only contribute to the two bottom lines proposed by the Chinese government of guaranteeing the country’s food security and not returning to poverty on a large scale, but also provide experiences and solutions from China for poor regions in the world facing food crises and problems with increasing farmers’ incomes.

### 4.2. Robustness Tests

#### 4.2.1. Robustness Test Dealing with Endogeneity

Although in the benchmark regression model, the fixed-effect model we used can reduce the interference of missing variables on the estimated results to a certain extent, there may still be potential endogeneity problems caused by bidirectional causality in this paper. The typical way to further deal with the endogeneity problem is to select instrumental variables that satisfy the two conditions of correlation and orthogonality. Considering the lagging nature of agricultural subsidies, that is, the agricultural subsidies have not yet been distributed to farmers when they cultivate grain in the first year, means that the agricultural subsidy obtained in the first year can only be put into cultivation in the next year. Therefore, we consider the core explanatory variable with a lag of one period as the first instrumental variable. In addition, given that the instrumental variable should be related to the endogenous explanatory variable and independent of the random error term, we select provincial general public budget expenditure as the second instrumental variable, which is a direct reflection of the provincial fiscal expenditure. Although the agricultural subsidy policy is implemented by the central government, the standards, methods, and foundation of the implementation are determined by the local government taking into account the actual situation of the province. In particular, the subsidy standard is largely affected by the provincial fiscal expenditure level. Generally, when the level of fiscal expenditure is higher, the corresponding agricultural subsidy standard is also higher, and under the same conditions, farmers will receive more agricultural subsidies. However, the level of government fiscal expenditure does not affect the farmers’ capacity of grain production or income, both the correlation and exogenous conditions are satisfied, and thus, it can be selected as an instrumental variable. 

Table 3 depicts the regression results of agricultural subsidy policy effectiveness using the instrumental variables. First, we estimated the econometric model by the two-stage least squares (2SLS) method. The regression results obtained after solving the endogeneity problem are shown in Columns (1)–(3) of Table 3, which indicate that agricultural subsidies had a positive impact on the total sown area, total output, and total income at the 10% significance level or better. Moreover, the results of the three statistics in the table demonstrate that the instrumental variables selected in this paper did not have the problems of under-identification, weak instruments, or over-identification. In addition, we adopted the limited information maximum likelihood (LIML) estimation method for robustness testing. According to the results of Columns (4)–(6) in Table 3, compared with the 2SLS regression results, neither the signs of the coefficients nor the significance level change, and the influence of agricultural subsidies is consistent, which reveals that the conclusions of this paper are not affected by the endogeneity problem.

#### 4.2.2. Substitution and Combination of Variables

Numerous studies propose that the selection of inappropriate variables has a severe effect on the estimation results of econometric models. Therefore, we reconsider the empirical test from the perspective of replacing dependent variables and combining related explanatory variables to further test the robustness of the conclusions obtained in this paper. (1) Replacement of the dependent variables. The explained variable in the benchmark regression adopts the concept of grain in a broad sense, whereas in the robustness test, the explained variable is replaced by the narrow concept of grain. Concretely speaking, the concept of grain in a narrow sense usually refers to wheat, corn, and rice. These three kinds of grain are regarded as the three major food crops in China, and their sown area accounts for 75% of China’s total area of grain, which has high representativeness. Consequently, the total sown area, total yield, and total income of the three major kinds of grain were selected as the dependent variables for the robustness test. (2) Combination of related control variables. In this part, we combined two variables, whether households can borrow money from either the private sector or financial institutions, into one, denoted as *loan*, which was introduced into the model as a new control variable, and then re-estimated the effect of the agricultural subsidy policy.

Table 4 demonstrates the regression results of the effect of the agricultural subsidy policy after substituting and combining the variables. In particular, Columns (1)–(3) in Table 4 reveal the regression results of the effect of the agricultural subsidy policy after replacing the dependent variables. Moreover, the effect of the agricultural subsidy policy is shown in Columns (4)–(6), which list the regression results of simultaneously replacing the dependent variables and combining the control variables. Columns (1)–(6) clearly show that whether only the dependent variables are replaced or both the dependent variables are replaced and the control variables are combined, it is evident that agricultural subsidies always have a significant effect on the total sown area, total output, and total income of farmers, which is consistent with the benchmark regression results.

## 5. Extended Analysis

### 5.1. Heterogeneity Analysis

#### 5.1.1. Heterogeneity Analysis Based on Geographic Conditions: Differences between Northern and Southern Regions

The provinces involved in the survey are located in various regions of China, with great differences in natural conditions, such as topography, climate, and precipitation, which may lead to heterogeneity in the effect of the agricultural subsidy policy. Therefore, based on the Qinling–Huaihe boundary, we classified the five provinces involved in the survey into northern and southern regions and examined the variation in the effectiveness of the agricultural subsidy policy. Specifically, we classified Gansu, Henan, and Shandong provinces as northern regions, while we classified Hunan and Sichuan provinces as southern regions.

Table 5 demonstrates the different effects of the agricultural subsidy policy between the southern and northern regions. According to the regression results, in the southern regions, agricultural subsidies significantly enhanced total sown area but had no obvious impact on total grain output or total income. In contrast, in the northern regions, the agricultural subsidy policy had positive effects, whether on total sown area, total grain output, or total income at the 1% significance level. There are several possible reasons for this phenomenon. In the northern regions, there is more arable land, and the terrain is relatively flat; thus, the arable land is more concentrated. However, in the southern regions, the terrain is mostly hilly, and thus, the cultivated land is correspondingly more scattered. This means that the northern regions have an advantage over the southern regions in terms of resource endowment in planting grain. Agricultural subsidies noticeably raise the enthusiasm of farmers in the northern regions to cultivate grain, and consequently, the effect of the agricultural subsidy policy is more obvious in these regions.

#### 5.1.2. Heterogeneity Analysis Based on Economic Development Level: Differences in Defining Standards of Poor Counties

The 10 target counties in the survey contain both state-level and non-state-level poverty-stricken counties. Given the significant differences in the level of economic development between the two types of counties, the effect of the agricultural subsidy policy was also expected to differ. The national poverty-stricken counties were announced by the central government in 2001, with a total of 592 counties initially. Eleven years later, China adjusted the list, removing 38 counties and adding 38 counties, and the data used in this paper do not come from the removed or added counties mentioned above. Therefore, the classification standard of the national poverty-stricken counties adopted in this study comes from the list of poverty-stricken counties announced in 2012. In particular, the state-level poverty-stricken counties include Nanjiang, Xin, Sangzhi, Huayuan, Longxi, and Jingning, and the non-state-level poverty-stricken counties include Xichong, Yuanyang, Sishui, and Yiyuan.

The estimation results produced after dividing all the sample counties into state-level and non-state-level poverty-stricken counties are provided in Table 6. It can be observed that the relationship between the agricultural subsidy policy and the total sown area in non-state-level poverty-stricken counties is highly significant, but the policy had a weak impact on the total grain yield and total income of farmers. By contrast, in the state-level poverty-stricken counties, agricultural subsidies had a significant positive impact on the total output and total income of farmers in addition to the total sown area. This phenomenon can be explained by the following points. On the one hand, farmers in state-level poverty-stricken counties rely more on the land. Therefore, subsidizing these farmers stimulates their enthusiasm for cultivation, expands the total sown area, and consequently plays a positive role in grain production and farmers’ income. On the other hand, compared with farmers in national poverty-stricken counties, farmers in non-state-level poverty-stricken counties have better economic conditions and depend less on the land, and the promotion effect of the agricultural subsidy policy is relatively weak.

#### 5.1.3. Heterogeneity Analysis Based on Functional Orientation of Grain Production: Differences in Distribution of Crop Production Areas

There are 13 major grain producing areas in China, accounting for more than 75% of the national grain output and with possession of 71% of the national grain inventory. Therefore, we divide all the provinces investigated into two categories based on whether they are major grain producing areas to explore the differences in the effectiveness of the agricultural subsidy policy. Notably, Shandong, Henan, Sichuan, and Hunan are main grain producing areas, while Gansu is not.

The estimation results in Table 7 indicate that the effects of the agricultural subsidy policy vary after distinguishing between major grain producing areas and non-major grain producing areas. It is clear that in non-major grain producing areas, only the relationship between agricultural subsidies and total sown area is significantly positive. Nevertheless, in major grain producing areas, agricultural subsidies have a positive impact on not only the total sown area but also the total grain yield and total income of farmers. The reason for this phenomenon may be that, compared with those in non-major grain producing areas, farmers in main grain producing areas enjoy better cultivation conditions. Therefore, agricultural subsidies in major grain producing areas can more effectively mobilize farmers to plant grain, enlarge the sown area, and ultimately stimulate the improvement of grain production and farmers’ income.

### 5.2. Mechanism Analysis

From the perspective of farmers’ willingness to engage in cultivation, we examine the role of the proportion of idle land on the effect of agricultural subsidy policy. The regression results regarding the proportion of idle land as an intervening variable are provided in Table 8. Accordingly, Column (1) of Table 8 shows that agricultural subsidies are negatively related to the proportion of idle land, passing the 10% significance test, indicating that subsidizing farmers can effectively avoid the phenomenon of land abandonment and mobilize farmers to cultivate grain. Furthermore, the estimation results of Columns (2)–(4) depict that both the proportion of idle land and agricultural subsidies have a significant impact on the total sown area, total output, and total income of farmers at the 5% significance level or better, suggesting that the proportion of idle land acts as a mediating variable in the process of agricultural subsidies affecting farmers’ capacity of grain production and income. Agricultural subsidies boost farmers’ willingness to cultivate the land and reduce the proportion of idle land in their self-owned arable land so that the total sown area, total output, and total income of farmers increase constantly. Meanwhile, the coefficient and confidence interval of the mediation effect under the Bootstrap method are also shown in Table 8. The coefficient of the mediation effect is significant at the 1% statistical level, and the confidence interval is significantly different from 0, which proves the robustness of the mediation effect tested in this study. Influence 2 of this paper is confirmed.

## 6. Conclusions and Suggestions

This paper derived the effects of the agricultural subsidy policy under equilibrium conditions. These predictions were then tested using the tracking survey data of China’s poverty-stricken villages mutual aid fund project. The conclusions of this study are proposed as follows. First, agricultural subsidies have a significant positive impact on the total sown area, total output, and total income of farmers at the 5% significance level or better, indicating that subsidizing agricultural production is conducive to improving farmers’ capacity of grain production and income, which is consistent with the inferences of the theoretical model. Second, due to the differences in geographic conditions, economic development level, and function orientation of grain production in various regions, the effects of agricultural subsidies are diverse, with different degrees of influence on the total sown area, total grain output, and total income of farmers. Third, agricultural subsidies can effectively raise farmers’ willingness to cultivate grain and reduce the proportion of idle land, thereby improving farmers’ total sown area, total grain output, and total income. Notably, farmers’ willingness to engage in cultivation is an important mechanism influencing the effectiveness of the agricultural subsidy policy. 

Based on the above conclusions, the following policy recommendations and measures are put forward. First, the government should guarantee the implementation of the food security policy, optimize food production and operation modes, improve the ability to prevent and control natural disasters, provide sufficient food for the market, and stabilize domestic food prices. Second, the government should pay more attention to land management projects, deepen the implementation of the strategy of “increasing grain production through soil conservation,” and protect arable land as the basis of grain production, which is conducive to ensuring the steady increase in grain production and simultaneously augmenting farmers’ income. Third, farmers, as the main body of agricultural production, should develop intensive and modern agriculture based on their conditions, local advantages, and agricultural subsidies; promote self-sufficiency in grain supply; and strengthen their dominant position in the food industry chain, contributing to the advancement of national food security. Fourth, boosting food production and ensuring national food security is a systematic project requiring more attention be paid to the diversity and synergy of subsidy policies and the implementation of targeted agricultural subsidy policies for different regions to release the combined effect.

## Figures and Tables

**Table 1:** Main variables and their descriptive statistics. (*n* = 3348).

Variable	Name	Definition	Std	Mean	Mini	Max
Dependent variables						
area	Sown area	Mu/household	18.095	8.257	0	608.8
output	Total output of grain	Kilogram/household	3858.112	2885.069	0	90000
income	Total income of grain	Yuan/household	8164.047	5802.065	0	194600
Independent variable						
subsidy	Agricultural subsidies	Yuan/household	459.247	364.3711	0	8061
Control variables						
age	Head of household’s age	Year	11.209	54.470	18	94
education	Head of household’s education	1–5 = no schooling, primary school, junior high school, high school, and university, respectively	0.879	2.362	1	5
healthy	Head of household’s health status	1 = healthy, 0 = unhealthy	0.500	0.521	0	1
cadre	Head of household’s cadre status	1 = yes, 0 = no	0.245	0.064	0	1
labour	Family’s labor force size	Person/household	1.231	2.738	0	9
work	Whether the family members go out as migrant workers	1 = family members obtain information about going out to work, 0 = no	0.428	0.240	0	1
assets	Value of household production and operation fixed assets	Yuan/household	24915.014	4286.507	0	600420
gift	Expenditure of household gift-giving for the favor pattern	Yuan/household	4099.223	1808.213	0	80000
P-loan	Whether family can borrow money from private sector	1 = yes, 0 = no	0.436	0.744	0	1
B-loan	Whether family can gain access to loans from financial institutions	1 = yes, 0 = no	0.442	0.266	0	1
village	Whether village is credit village assessed by credit cooperatives	1 = yes, 0 = no	0.484	0.625	0	1
meetings	Frequency of “two rural committee meetings”	Times/year	4.358	5.737	0	36
Mechanism variables						
Proportion	Proportion of idle land	—	0.143	0.037	0	1

Data source: Personal statistics.

**Table 2:** Benchmark regression results.

Variable	Ln(Area)		Ln(Output)		Ln(Income)	
	(1)	(2)	(3)	(4)	(5)	(6)
Ln(subsidy)	0.060 ***	0.055 ***	0.116 ***	0.098 ***	0.097 ***	0.077 **
	(0.0102)	(0.0096)	(0.0276)	(0.0260)	(0.0321)	(0.0304)
age		0.010 ***		0.027 **		0.029 **
		(0.0032)		(0.0107)		(0.0115)
education		0.026		0.028		0.008
		(0.0254)		(0.0672)		(0.0781)
healthy		0.074 **		0.043		0.065
		(0.0299)		(0.0740)		(0.0862)
cadre		0.106		−0.077		−0.166
		(0.0861)		(0.2022)		(0.2241)
labour		0.060 ***		0.220 ***		0.225 ***
		(0.0134)		(0.0392)		(0.0441)
work		0.029		0.092		0.057
		(0.0293)		(0.0776)		(0.0852)
Ln(assets)		0.052 *** (0.0063)		0.123 *** (0.0210)		0.144 *** (0.0241)
Ln(gift)		0.014 **		0.031 *		0.043 **
		(0.0061)		(0.0160)		(0.0175)
P-loan		0.031		0.050		0.115
		(0.0313)		(0.0777)		(0.0887)
B-loan		0.016		−0.106		−0.089
		(0.0307)		(0.0862)		(0.0986)
village		0.104 ***		−0.001		0.066
		(0.0389)		(0.1061)		(0.1230)
meetings		−0.002		0.009		0.014
		(0.0035)		(0.0097)		(0.0108)
Constant term	1.361 ***	0.134	6.656 ***	3.662 ***	7.219 ***	3.819 ***
	(0.0495)	(0.2052)	(0.1311)	(0.6979)	(0.1511)	(0.7582)
Year fixed effects	yes	yes	yes	yes	yes	yes
Area fixed effects	yes	yes	yes	yes	yes	yes
Observations	3348	3348	3348	3348	3348	3348
R-squared	0.029	0.093	0.023	0.084	0.019	0.080

Note: Robust standard errors in parentheses; *** *p* < 0.01, ** *p* < 0.05, * *p* < 0.1.

**Table 3:** Regression results obtained by instrumental variable method.

Variable	2SLS			LIML		
	Ln(Area)	Ln(Output)	Ln(Income)	Ln(Area)	Ln(Output)	Ln(Income)
	(1)	(2)	(3)	(4)	(5)	(6)
Ln(subsidy)	0.359 ***	0.418 ***	0.291*	0.359 ***	0.421 ***	0.293 *
	(0.0582)	(0.1305)	(0.1495)	(0.0583)	(0.1318)	(0.1564)
Control variables	yes	yes	yes	yes	yes	yes
Constant term	−0.431 **	4.176 ***	5.362 ***	−0.603 ***	3.782 ***	4.955 ***
	(0.1893)	(0.4596)	(0.5202)	(0.2017)	(0.4994)	(0.5654)
Kleibergen–Paaprk LM	60.789 (0.0000)	60.789 (0.0000)	60.789 (0.0000)			
Kleibergen–Paaprk Wald F	29.176 (19.930)	29.176 (19.930)	29.176 (19.930)			
Hansen J	0.115 (0.7345)	0.827 (0.3632)	1.283 (0.2574)			
Year fixed effects	yes	yes	yes	yes	yes	yes
Area fixed effects	yes	yes	yes	yes	yes	yes
Observations	2232	2232	2232	2232	2232	2232
R-squared	0.344	0.209	0.176	0.344	0.208	0.176

Note: Robust standard errors in parentheses; *** *p* < 0.01, ** *p* < 0.05, * *p* < 0.1. The *p*-values of Kleibergen–Paaprk LM and Hansen J for under-identification and over-identification tests are in parentheses. The critical values at the 10% level for the Stock–Yogo weak identification test of the Kleibergen–Paaprk Wald F statistic are also in parentheses.

**Table 4:** Estimated results for substituting and combining variables.

Variable	Replacing Dependent Variables			Replacing Dependent Variables and Combining Control Variables		
	Ln(Area)	Ln(Output)	Ln(Income)	Ln(Area)	Ln(Output)	Ln(Income)
	(1)	(2)	(3)	(4)	(5)	(6)
Ln(subsidy)	0.048 ***	0.096 ***	0.070 **	0.048 ***	0.094 ***	0.069 **
	(0.0092)	(0.0280)	(0.0300)	(0.0092)	(0.0279)	(0.0298)
loan				−0.002	−0.049	0.024
				(0.0289)	(0.0860)	(0.0918)
Constant term	0.145	4.040 ***	3.914 ***	0.157	4.122 ***	3.984 ***
	(0.1905)	(0.7356)	(0.7411)	(0.1871)	(0.7262)	(0.7331)
Year fixed effects	yes	yes	yes	yes	yes	yes
Area fixed effects	yes	yes	yes	yes	yes	yes
Observations	3348	3348	3348	3348	3348	3348
R-squared	0.076	0.077	0.066	0.075	0.076	0.065

Note: Robust standard errors in parentheses; *** *p* < 0.01, ** *p* < 0.05.

**Table 5:** Estimated results of distinguishing northern and southern regions.

Variable	Southern Regions			Northern Regions		
	Ln(Area)	Ln(Output)	Ln(Income)	Ln(Area)	Ln(Output)	Ln(Income)
	(1)	(2)	(3)	(4)	(5)	(6)
Ln(subsidy)	0.487 *	−0.033	−0.003	0.047 ***	0.111 ***	0.092 ***
	(0.0262)	(0.0687)	(0.0725)	(0.0098)	(0.0266)	(0.0321)
Control variables	yes	yes	yes	yes	yes	yes
Constant term	0.079	3.893	3.158 **	0.598 ***	4.714 ***	4.577 ***
	(0.5420)	(1.3299)	(1.3570)	(0.1989)	(0.6188)	(0.7192)
Year fixed effects	yes	yes	yes	yes	yes	yes
Area fixed effects	yes	yes	yes	yes	yes	yes
Observations	1212	1212	1212	2136	2136	2136
R-squared	0.098	0.099	0.100	0.093	0.092	0.075

Note: Robust standard errors in parentheses; *** *p* < 0.01, ** *p* < 0.05, * *p* < 0.1.

**Table 6:** Regression results of distinguishing state-level and non-state-level poverty-stricken counties.

Variable	Non-State-Level Poverty-Stricken Counties			State-Level Poverty-Stricken Counties		
	Ln(Area)	Ln(Output)	Ln(Income)	Ln(Area)	Ln(Output)	Ln(Income)
	(1)	(2)	(3)	(4)	(5)	(6)
Ln(subsidy)	0.027 **	0.012	−0.034	0.067 ***	0.152 ***	0.175 ***
	(0.0125)	(0.0275)	(0.0360)	(0.0141)	(0.0424)	(0.0454)
Constant term	0.691 ***	5.530 ***	5.266 ***	0.499 *	3.946 ***	3.777 ***
	(0.2555)	(0.7254)	(0.8676)	(0.2689)	(0.8938)	(0.9394)
Control variables	yes	yes	yes	yes	yes	yes
Year fixed effects	yes	yes	yes	yes	yes	yes
Area fixed effects	yes	yes	yes	yes	yes	yes
Observations	1461	1461	1461	1887	1887	1887
R-squared	0.086	0.053	0.056	0.090	0.105	0.103

Note: Robust standard errors in parentheses; *** *p* < 0.01, ** *p* < 0.05, * *p* < 0.1.

**Table 7:** Regression results of distinguishing major and non-major grain producing areas.

Variable	Non-Major Grain Producing Areas			Major Grain Producing Areas		
	Ln(Area)	Ln(Output)	Ln(Income)	Ln(Area)	Ln(Output)	Ln(Income)
	(1)	(2)	(3)	(4)	(5)	(6)
Ln(subsidy)	0.038 *	0.056	0.085	0.050 ***	0.085 ***	0.059 *
	(0.0198)	(0.0591)	(0.0625)	(0.0109)	(0.0282)	(0.0338)
Control variables	yes	yes	yes	yes	yes	yes
Constant term	0.433	3.383 ***	3.399 ***	0.744	5.729 ***	5.397 ***
	(0.3092)	(1.0810)	(1.1304)	(0.2199)	(0.7325)	(0.8251)
Year fixed effects	yes	yes	yes	yes	yes	yes
Area fixed effects	yes	yes	yes	yes	yes	yes
Observations	1287	1287	1287	2061	2061	2061
R-squared	0.096	0.100	0.102	0.081	0.065	0.057

Note: Robust standard errors in parentheses; *** *p* < 0.01, * *p* < 0.1.

**Table 8:** Regression results of mediating effect.

Variable	Proportion	Ln(Area)	Ln(Output)	Ln(Income)
	(1)	(2)	(3)	(4)
Proportion		−0.426 *** (0.0832)	−0.817 *** (0.2420)	−0.706 ** (0.2742)
Ln(subsidy)	−0.002 *	0.089 ***	0.147 ***	0.116 ***
	(0.0015)	(0.0091)	(0.0232)	(0.0263)
Constant term	0.018	−0.212*	4.383 ***	5.087 ***
	(0.0221)	(0.1233)	(0.3450)	(0.3821)
Year fixed effects	yes	yes	yes	yes
Area fixed effects	yes	yes	yes	yes
Coefficient of Bootstrap method		0.178 *** (0.0082)	0.158 *** (0.0191)	0.082 *** (0.0225)
Confidence interval of bootstrap method		[0.162,0.193]	[0.122,0.193]	[0.037,0.125]
Observations	3348	3348	3348	3348
R-squared	0.018	0.092	0.079	0.072

Note: Standard errors in parentheses; *** *p* < 0.01, ** *p* < 0.05, * *p* < 0.1.

## Data Availability

The data will be available upon request.
